# Molecular Signaling Pathways, Regulatory and Coactivator Networks, and Emerging Mechanisms in Hepatocellular Carcinoma

**DOI:** 10.3390/biomedicines14092046

**Published:** 2026-09-11

**Authors:** Rohit K. Srivastava, Pratibha Singh, David M. Lonard

**Affiliations:** Department of Molecular and Cellular Biology, Baylor College of Medicine, Houston, TX 77030, USA; pratibha.singh@bcm.edu

**Keywords:** liver cancer, cancer stem cells, immune evasion, epigenetic reprogramming, therapeutic resistance, precision medicine

## Abstract

Hepatocellular carcinoma (HCC) is the most common primary liver malignancy and a leading cause of cancer-related mortality worldwide. Despite advances in diagnosis and therapy, the prognosis for advanced HCC remains poor due to late-stage diagnosis, high recurrence rates, therapeutic resistance, and pronounced molecular heterogeneity. HCC development is driven by complex somatic gene alterations, epigenetic reprogramming, dysregulated signaling pathways, metabolic changes, and an immunosuppressive tumor microenvironment. Molecular profiling studies have identified key oncogenic pathways involved in HCC progression, including MAPK/ERK (mitogen-activated protein kinase/extracellular signal-regulated kinase), Wnt/β-catenin, PI3K/AKT/mTOR (Phosphoinositide 3-kinase/Protein Kinase B/mechanistic Target of Rapamycin), Hippo-YAP/TAZ, (Yes-associated protein/transcriptional co-activator with PDZ-binding motif) cell cycle regulators, and p53-mediated tumor suppression. These pathways coordinate critical cellular processes such as proliferation, survival, metabolism, invasion, and genomic stability. Emerging mechanisms, including cancer stem cell plasticity, immune evasion, epigenetic dysregulation, and steroid receptor coactivator (SRC)-dependent transcriptional regulation, further contribute to tumor progression and therapeutic resistance. Additionally, recent bioinformatic analyses suggest a potential role for progesterone-mediated oocyte maturation pathways in HCC, although their functional relevance remains unclear. A thorough understanding of these interconnected mechanisms could lead to novel therapeutic targets and the development of more effective, personalized treatment strategies for HCC. This review discusses key signaling pathways and emerging mechanisms in HCC and their roles in disease development and treatment.

## 1. Introduction

Cancer is the second most common cause of death worldwide after cardiovascular diseases [1,2,3,4]. For all cancers in the United States, the 5-year relative survival was 70% for patients diagnosed during 2015–2021, with survival of 69% for regional-stage disease and 35% for distant-stage disease [5]. Liver cancer ranks fourth in common cause of cancer death worldwide, with an estimated incidence of >1 million cases by 2025 [6]. HCC is the most commonly occurring subtype of liver cancer and accounts for ~90% of cases [7]. Approximately two-thirds of patients are diagnosed at an advanced stage, which substantially limits available treatment options. Moreover, despite advances in current therapeutic strategies, HCC is characterized by a high rate of tumor recurrence following surgical resection. The major risk factors associated with HCC include chronic hepatitis B or C virus infection, exposure to aflatoxin B1, excessive alcohol consumption, and alcohol-associated liver disease [8]. HCC is a complex multistep biological process that includes genetic and epigenetic alterations [9]. However, there has been significant progress in our understanding of the key oncogenic and tumor suppressor mechanisms involved in HCC [10]. Identification of dysregulated cellular signaling networks has provided important insights into HCC pathogenesis and offers opportunities for the development of novel therapeutic strategies.

In recent years, significant progress has been made in the understanding of the molecular mechanisms involved in HCC. Several signaling pathways have been dysregulated and have been identified, suggesting their crucial roles in HCC development [11]. Well-established pathways, including MAPK/ERK, Wnt/β-catenin, Hippo signaling, and cell cycle dysregulation, have been extensively studied. Several recent reviews have summarized these major oncogenic pathways and their relevance to targeted therapy and immunotherapy. The current review expands on these studies by reviewing established signaling pathways and less commonly discussed regulatory mechanisms with a particular emphasis on steroid receptor coactivators (SRCs). SRC family members, particularly SRC-1 and SRC-3, are involved in transcriptional programs associated with HCC growth, invasion, metabolism and therapeutic resistance. Moreover, this review also discusses cancer stem cell plasticity, immune evasion, epigenetic dysregulation, and the newly identified progesterone-mediated oocyte maturation pathway in HCC pathway-enrichment studies. Thus, this review is different from previous pathway-oriented reviews of HCC by integrating on established signaling pathways, SRC-dependent transcriptional regulation, and emerging mechanisms.

## 2. Major Oncogenic Signaling Pathways and Molecular Regulators Driving HCC

### 2.1. MAPK/ERK Signaling Cascade in HCC

The MAPK/ERK signaling pathway is frequently mutated in human cancers and plays a critical role in tumorigenesis [12]. A RAS family gene is reported to be mutated in approximately 20–30% of all human solid tumors [13]. However, the role of the MAPK/ERK pathway in human HCC has been largely ignored, mainly because RAS (Rat Sarcoma)and RAF (rapidly accelerated fibrosarcoma) mutations are rare and are found in less than 5% of cases of HCC [14]. The incidence of mutations in the different parts of the MAPK/ERK signaling pathway is low, but frequent activation of this signaling pathway has been reported in HCC patients [15]. Moreover, poor survival rates were observed in HCC patients with high levels of activated RAS effectors, indicating the importance of MAPK/ERK signaling for prognosis [16]. Therefore, although higher activation of the MAPK/ERK pathway correlates with worse survival in HCC, a standardized pathway specific 2- or 5-year survival rate has not been defined for direct comparison with the overall HCC population. Consistent with this, it has been suggested that overexpression of RAF-1 is an independent predictor of early recurrence of the tumor and poor prognosis [17]. Also consistent with these findings, activation of the MAPK/ERK signaling cascade is seen in the majority of human HCCs when RAS, RAF, and downstream components are present in wild-type forms [18]. The frequent activation of the MAPK/ERK signaling pathway in HCC cannot be fully explained by RAS and RAF mutations, and other possibilities include the aberrant activation of upstream growth factors and receptors, the downregulation of negative regulators of the MAPK/ERK signaling pathway, or the upregulation of its positive modulators. The MAPK/ERK signaling pathway can be indirectly activated by either suppressing the regulators that oppose the RAS-RAF-MEK-ERK cascade or activating the modulators that strengthen the signaling pathway. Several growth factors, such as EGF (epidermal growth factor) and TGF-β (transforming growth factor-beta), have been found to be involved in the activation of the MAPK/ERK signaling pathway in liver cancer, especially in the early stage [19], suggesting that MAPK/ERK may be involved in hepatocyte neoplastic transformation. In HCC, hepatocyte growth factor (HGF), normally secreted by mesenchymal cells, is instead released by non-parenchymal hepatic stellate cells (HSC) and then binds to the HGFR (c-Met receptor) receptor on the surface of hepatocytes and initiates the MAPK/ERK signaling pathways [20]. In addition, some non-coding RNAs, such as miR-4510, miR-30a, miR-330-5p, miR-487, and long non-coding RNAs (lncRNAs), can also regulate the MAPK/ERK signaling pathway in HCC. In HCC, miR-4510 and miR-30a are down-regulated, while miR-330-5p and miR-487 are up-regulated [21,22]. Whereas some lncRNAs, such as BRAF-activated non-coding RNA, IGF2AS, LL22NC03-N14H11.1, and URHC, on the other hand, are upregulated in HCC and have been shown to promote tumorigenesis in the liver by activating the MAPK/ERK signaling [23,24,25]. On the contrary, lncRNAs RUNX1-IT1 and CASC2 inhibit the MAPK/ERK signaling pathway, and they are significantly down-regulated in HCC [26,27]. Various MAPK inhibitors have been developed and are summarized in Table 1.

### 2.2. Wnt/β-Catenin Signaling and Transcriptional Activation in HCC

Dysregulated Wnt/β-catenin signaling is responsible for precancerous dysplasia, malignant change in liver cells, and malignant growth of cancer cells [28]. Gain of function mutations in *CTNNB1*, the gene encoding β-catenin, and loss of function mutations of *AXIN1* (axis inhibition protein) have been identified in about 35% of human HCC samples [29]. However, somatic mutations in exon 3 of CTNNB1 can affect serine/threonine phosphorylation sites or adjacent amino acids, resulting in reduced β-catenin phosphorylation and degradation. This phenomenon causes the stabilization and nuclear translocation of β-catenin [29].

Human HCCs with Wnt/β-catenin pathway activation show different gene expression signatures and clinical, pathological, and molecular features. Several studies indicate that β-catenin overexpression and mutations are more frequent in HCV (hepatitis C virus)-related than in HBV (hepatitis B virus)-related HCCs [30] and are frequently detected in HCCs with noncirrhotic liver without typical risk factors for HCC [31,32]. Activation of the Wnt/β-catenin pathway has been associated with early HCC and tumor development [33]. In addition, activated Wnt/β-catenin has been reported to interact with several signaling pathways to promote the development of HCC, and the interaction is through its downstream effectors [30].

However, the presence of mutations in components of the WNT/β-catenin pathway cannot fully account for the high frequency of β-catenin signaling activation in HCC. Abnormal activation of WNT/β-catenin in HCC is reported to be caused by epigenetic modifications, such as DNA methylation [34], histone remodeling [35], or non-coding RNA [36]. In fact, 9–12.5% of HCC patients exhibited Met overexpression/activation and β-catenin mutations, and subsequent studies indicated that co-expression of hMet and mutant β-catenin genes induces the formation of liver tumors in rodents [37].

Liver cancer cells overproduce Wnt ligands, which induce the infiltration of macrophages and other cell types into the tumor microenvironment [28]. On the other hand, Wnt inhibitors such as SFRP1/4/5 and Kallistatin are decreased in HCCs, allowing Wnt molecules to interact with receptors [38]. However, the strongly up-regulated miR-1246 decreases AXIN2 and GSK3 (glycogen synthase kinase) expression [39]. APC (adenomatous polyposis coli) gene hypermethylation causes loss of APC protein [40]. Collectively, these epigenetic changes impair the destruction complex to promote Wnt/β-catenin signaling in HCCs. Even though transgenic mice were used to study the oncogenic function of Wnt/β-catenin mutations in HCC, some studies indicate that transgenic mice overexpressing activated mutant forms of β-catenin develop hepatotoxicity but not HCC [41]. These results suggest that Wnt/β-catenin activation by itself may not be sufficient to drive liver cancer progression. To cause HCC growth, a second signal must work in tandem with activated β-catenin. Recent hydrodynamic transfection studies in mice have shown that oncogenic β-catenin can work together with other cancer-promoting genes, including c-Met (mesenchymal–epithelial transition factor), *K-Ras*V12, activated AKT, LKB1 (liver kinase B1), and Nrf2 (nuclear factor erythroid 2–related factor 2), to promote HCC development [42].

Moreover, the WNT/β-catenin signaling pathway is associated with the state of the tumor microenvironment [43]. Many studies have found that autophagy plays an important role in the tumor microenvironment. Autophagy is a highly conserved cellular process that plays an important role in both normal liver function and liver disease [44,45]. The relation of autophagy to metabolism is, however, not known. Recently, it has been shown that the activation of autophagy inhibited the proliferation of HepG2 cells by inhibiting GPC3/WNT/β-catenin signaling [46]. Additionally, autophagy promotes metastasis and glycolysis in HCC cells by activating WNT/β-catenin signaling, which induces MCT1 (Monocarboxylate transporter 1) expression, a key factor in lactic acid transport and H^+^ clearance in cancer cells [47]. Recent studies have shown that Wnt/β-catenin signaling and abnormal regulation of the proton pump (v-ATPase) are responsible for HCC, and the pharmacological target of v-ATPase/autophagy inhibits the growth of HCC cells [43,48].

Despite strong evidence linking Wnt/β-catenin activation to HCC, its effects may vary depending on the genetic alterations and signaling pathways present in the tumor. Activation of β-catenin alone is not sufficient to induce HCC in some experimental models [49], whereas tumor formation occurs when β-catenin activation is combined with additional oncogenic signals such as c-Met, KRAS, or AKT [50]. The results suggest that Wnt/β-catenin signaling is not the sole driving force in HCC. An important remaining question is which genetic changes or signaling pathways determine whether β-catenin activation promotes tumor development, tumor progression, or resistance to treatment.

### 2.3. PI3K/AKT/mTOR Signaling and Metabolic Regulation in HCC

The PI3K/AKT/mTOR signaling pathway is a major oncogenic pathway in the development of HCC. This pathway regulates several cell processes related to the development of cancer, such as cell-cycle progression, cell survival, proliferation, apoptosis, metabolism, motility, angiogenesis, lipid metabolism, autophagy, and epithelial–mesenchymal transition. The pathway is frequently activated upstream through receptor tyrosine kinases or insulin/insulin-like growth factor signaling, leading to activation of PI3K and conversion of PIP2 (phosphatidylinositol (4,5)-bisphosphate) to PIP3 [51,52]. PIP3 recruits AKT to the plasma membrane, where AKT is activated by phosphorylation. The activated AKT then goes on to regulate a multitude of substrates, such as mTOR complexes, GSK3, FOXO (forkhead boxO), CREB (cAMP response element binding protein), MDM2/p53 (Mouse Double Minute 2 homolog), and NF-κB (nuclear factor kappa-light chain enhancers of activated B cells) [53,54]. PTEN (phosphatase and tensin homolog deleted on chromosome 10) is an important negative regulator that converts PIP3 to PIP2 so that loss or reduction in PTEN function may lead to upregulated PI3K/AKT signaling [55].

PI3K/AKT/mTOR signaling dysregulation is frequent in HCC. Published reviews show that a high proportion of HCC cases show activation or over-expression of this pathway, with ~40–50% or almost 50% of HCCs showing up-regulation of the mTOR pathway [51]. Increased PIK3CA expression has been associated with HCC proliferation, decreased apoptosis, and poor prognosis. The AKT pathway has also been associated with aggressive tumor behavior, early recurrence, and poor prognosis in patients with liver cancer [51,56]. Furthermore, PI3K/AKT/mTOR signaling is associated with the metabolic phenotype of HCC [57]. It can promote glycolysis and the Warburg effect, in part through regulation of glucose transporters and glycolytic enzymes. It can also support lipid synthesis through mTORC1/SREBP-dependent regulation of lipogenic enzymes such as FASN and ACC. The pathway is also involved in glutamine metabolism, pyrimidine metabolism, oxidative metabolism, and metabolic interactions in the tumor microenvironment [51].

The therapeutic importance of this pathway has been studied in HCC, but the clinical outcomes related to its therapeutic inhibition are still inconsistent. Multikinase inhibitors used in HCC, such as sorafenib, lenvatinib, regorafenib, and cabozantinib, can affect receptor tyrosine kinase signaling upstream of the PI3K/AKT/mTOR pathway. In addition, drugs that directly target PI3K, AKT, or mTOR have also been studied and some are being evaluated in clinical trials [52]. However, single agent mTOR inhibition has not shown significant clinical benefit in advanced HCC. Everolimus failed to improve overall survival in patients with advanced HCC after sorafenib failure in the phase III EVOLVE-1 trial [58]. These findings suggest that PI3K/AKT/mTOR signaling is biologically relevant in HCC, but pathway inhibition alone may be insufficient due to feedback regulation, pathway crosstalk, adaptive resistance, and toxicity. Thus, PI3K/AKT/mTOR is now defined as a well-established and clinically relevant signaling pathway in HCC. However, therapeutic intervention of the pathway may require biomarker-guided selection and rational combination approaches instead of simple single-agent inhibition.

Thus, although PI3K/AKT/mTOR signaling is frequently activated in HCC, increased pathway activity does not always indicate that the tumor will respond to PI3K/AKT/mTOR-targeted treatment. The failure of everolimus as a single-agent treatment in advanced HCC suggests that other signaling pathways, feedback mechanisms, differences among tumors, and the lack of reliable biomarkers for selecting patients may limit the effectiveness of mTOR-targeted therapy. Future studies should determine whether specific groups of HCC patients are more likely to benefit from PI3K/AKT/mTOR-targeted therapy and whether combining this treatment with other therapies can improve treatment response.

### 2.4. Hippo-YAP/TAZ Signaling Axis in HCC

**Figure 1:**
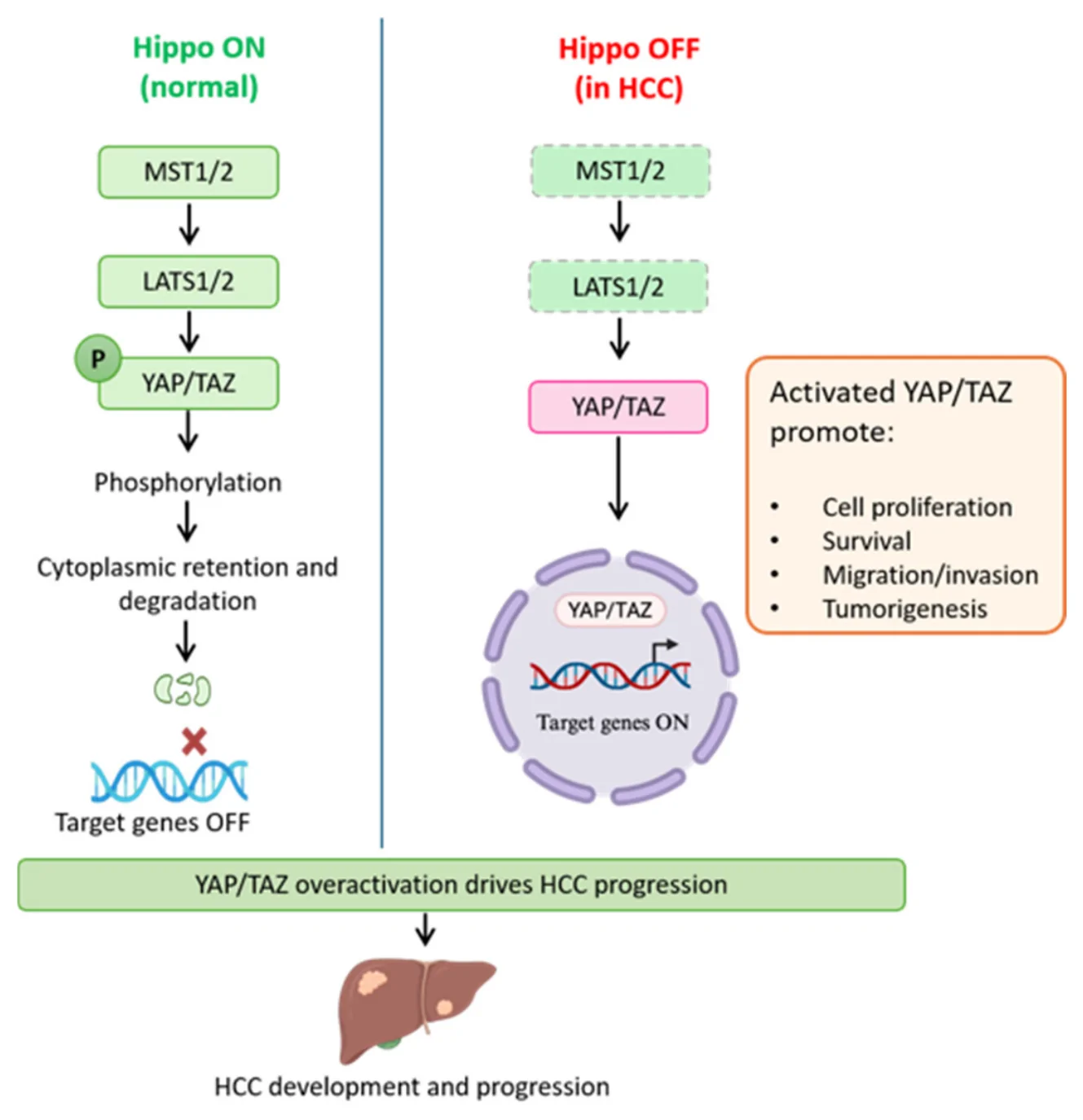
The Hippo signaling pathway in normal liver and HCC.

The Hippo signaling pathway plays an important role in controlling cell growth and has been linked to several diseases, including HCC. Its main components include MST1/2 (mammalian Ste20-like kinases ½), LATS1/2 (large tumor suppressor ½), YAP (Yes-associated protein), and TAZ (transcriptional co-activator with PDZ-binding motif), also known as WW domain-containing transcription regulator 1 (WWTR1) [59] (Figure 1).

One of the most important downstream effectors of the Hippo pathway is the transcriptional co-activator YAP, which is normally inhibited by the upstream kinases MST1/2 and LATS1/2. However, in HCC, YAP is often overexpressed and activated by mutations or other alterations in the components of the Hippo pathway, thus driving the survival and proliferation of cancer cells. The important oncogenic function of this protein was shown by the rapid growth of HCC after YAP overexpression [60]. Furthermore, in one study, the overexpression of YAP was shown to cause the development of HCC in a conditionally expressed YAP transgenic mouse model, which further implies a direct relationship between the dysregulation of the Hippo pathway and the development of liver tumors [61].

YAP activation occurs early during HCC development. Studies shown that YAP activation or overexpression occurs in roughly 54% to 62% (and up to 70% or more depending on nuclear accumulation markers) of HCC tumors [62]. However, mutations in the Hippo pathway are rare, and alternative regulatory mechanisms must exist to sustain YAP signaling. Many regulatory mechanisms have been identified in the search for upstream Hippo signaling regulators, including mechanotransduction, GPCR (G protein-coupled receptor) signaling, and interacting pathways, most notably the Wnt signaling pathway [63].

YAP and TAZ are key downstream effectors of the Hippo pathway. Like YAP, TAZ functions as a transcriptional co-activator and regulates genes involved in cell growth, differentiation, and survival [64,65]. TAZ dysregulation is also linked to the initiation and progression of several cancers, including HCC. TAZ induces the expression of genes including CTGF and AXL, which contribute to the growth, migration, and invasion of HCC cells [66,67]. TAZ can also reduce apoptosis in HCC cells by increasing the expression of anti-apoptotic proteins such as Bcl-2 and Bcl-xL [68]. Preclinical studies support the Hippo-YAP/TAZ pathway as a potential therapeutic target in HCC, although its clinical utility remains to be established. For example, therapies that activate Hippo pathway kinases or block YAP activity are currently being evaluated in preclinical studies and clinical trials [69]. Different approaches have been proposed, such as the development of TAZ inhibitors, RNA interference to silence TAZ expression, and modulation of TAZ upstream regulators such as the Hippo pathway kinases MST1/2 and LATS1/2 [70,71]. But further research is needed to determine whether these strategies are safe and effective in clinical practice. However, further studies are required to better understand the complex interactions between the Hippo pathway and HCC and to develop effective and safe treatments for this aggressive cancer.

### 2.5. Cell Cycle Deregulation and CDK-Mediated Control in HCC

Tumor cells show dysregulation of the cell cycle. Normal cells’ ability to enter a cell-cycle arrest after DNA damage is critical for maintaining genomic integrity [72]. Unregulated cell proliferation has emerged as a common factor for the development of all neoplastic diseases. The cell cycle is a highly regulated systematic process of cell growth. Change in the cell cycle machinery is one of the main mechanisms that change the normal cell proliferation process into an unregulated cell division state that eventually leads to carcinogenesis and cancer progression [73]. The human genome contains 21 genes encoding 20 cyclin-dependent kinases (CDKs), designated CDK1–CDK20, with CDK11 encoded by two separate genes, CDK11A and CDK11B [74,75]. CDKs are a family of serine/threonine kinases that play a pivotal role in the regulation of the cell cycle by phosphorylating target proteins involved in DNA replication and mitosis. CDK activity is regulated by association with specific cyclin partners, which promote their activation and substrate specificity. The family of CDKs genes is important for cell division and modulation of transcription [76].

**Figure 2:**
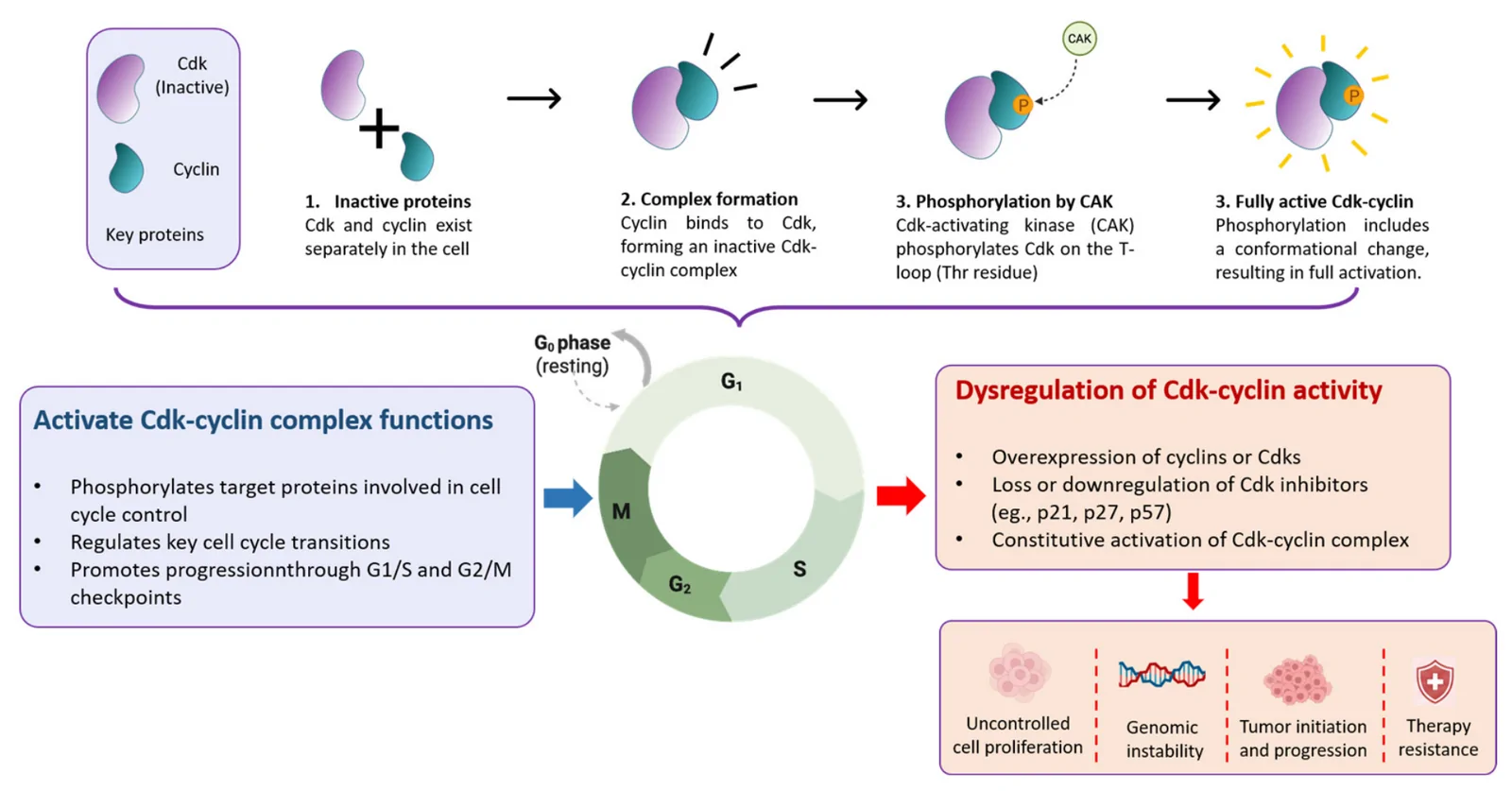
Activation, regulation, and dysregulation of cyclin–CDK complexes in HCC.

CDK activation occurs in two main steps. First, a cyclin binds to the CDK. The cyclin–CDK complex is then activated by phosphorylation of threonine 160 by cyclin-activating kinase (CAK) [74,77,78]. Phosphorylation is a reversible process that helps control enzyme activity. CAK activity is low in resting G0 cells and may be slightly higher in tumor cells than in normal cells [78,79]. In mammalian cells, this phosphorylation occurs only after cyclin binds to CDK (Figure 2).

CDK activity is often dysregulated in HCC where it is involved in cell cycle dysregulation by promoting cell proliferation and inhibiting apoptosis [80]. Specifically, CDK2 and CDK4 are involved in multiple cancer types including HCC development and progression [75,81]. CDK2 activity is essential for the G1/S transition and is frequently overexpressed in HCC, leading to uncontrolled cell proliferation [76]. CDK4 in complex with cyclin D1 participates in the progression of the G1 phase and is often overexpressed in HCC, which contributes to the loss of G1 checkpoint control and uncontrolled cell proliferation [75,82]. The tumor suppressor gene p16INK4a is an important negative regulator of CDK4 activity, and p16INK4a mutations and loss of p16INK4a function are common in HCC [83]. HCC often exhibits cyclin D1 overexpression, which may lead to CDK4 activity dysregulation [84]. Targeting dysregulated CDK activity in HCC is an attractive strategy for the development of novel therapeutics in liver malignancies. CDK inhibitors, including palbociclib, ribociclib, dinaciclib, and abemaciclib, have been investigated as potential anticancer agents, while preclinical studies have also demonstrated therapeutic activity of dinaciclib and the HDAC inhibitor panobinostat in hepatoblastoma [85,86,87,88]. In fact, growing data indicate that CDK overexpression may be involved in HCC tumorigenesis, progression, therapeutic effects, and outcomes [80]. However, further studies are needed to determine which CDKs have independent prognostic value in HCC and whether their broader application as cancer biomarkers can be extended to HCC prognosis.

### 2.6. p53-Mediated Tumor Suppressor Network in HCC

The tumor suppressor p53 and its other two members, p63 and p73, play an important role in the regulation of genomic stability and homeostasis. Alterations in the p53 pathway that regulates the cell cycle are found in most HCC patients [89]. In HCC, the p53 pathway can be altered by p53 mutations, loss of p14ARF activity, or increased levels of the p53 inhibitors MDM2 and MDM4 (MDMX) [89,90]. Activation of the p53 family is an important factor in DNA damage response, chemosensitivity, and prognosis of HCC. HCC intersects with apoptotic signaling pathways mediated by the p53 family at multiple levels [91]. TP53 alterations are common in tumors, but loss of both TP53 alleles is rare. This finding is a contradiction to the TP63 and TP73 genes, which are rarely lost or mutated in tumors. More than half of human tumors have a mutation in one TP53 allele, most commonly a missense mutation and less often a nonsense or frameshift mutation. In many cases the second TP53 allele is also lost, a process known as loss of heterozygosity (LOH) [92].

Several TP53 codons are mutated more frequently than others, especially codons 175, 245, 248, 249, 273, and 282, which are known as mutation “hot spots.” Mutations at codons 175, 245, 249, and 282 mainly affect p53 structure, whereas mutations at codons 248 and 273 directly affect DNA binding [93]. Together, these six hot-spot mutations account for more than 25% of the missense and nonsense mutations reported in the IARC (International Agency for Research on Cancer) TP53 database [94]. Hotspot mutants p53G245S are in the LOF (loss of function) group with a DNE (dominant-negative effect) on wild-type p53, while p53R175H, p53R248Q, and p53R273H have been shown to be GOF (gain of function) mutants. Based on the results of in vitro and in vivo studies performed in tissues or cells apart from the liver, p53R249S has been classified as a LOF mutation, whereas a GOF phenotype was observed in the liver and in the context of HCC [94].

Understanding how wild type p53 is inactivated in HCC may help in developing more personalized treatment strategies. In addition to p53 gene-based therapies, only a limited number of drugs targeting the p53 family have been evaluated in preclinical or clinical studies of HCC. TACE (transarterial chemoembolization), combined with the injection of recombinant adenovirus p53, was reported to be an effective therapy in unresectable HCC patients that contributed to improved overall and progression-free survival rates compared with TACE monotherapy alone [95].

Moreover, several small molecules able to recover the ability of p53 to regain its wild type function have been identified. The most significant of these is the linkage between p53 and MDM2. Nutlins are the first small molecules that directly target the p53/MDM2 interaction, which equalizes and accumulates p53. Nutlin-3 also induces apoptosis in HCC cells by directly activating p73 and preventing its interaction with MDM2 [96]. Another small molecule, RITA (reactivation of p53 and induction of tumor cell apoptosis), binds the N-terminus of p53 and specifically targets the p53/MDM4 interaction, preventing MDM2 from interacting with p53 [97]. Other examples are the spiro-oxindole MI-219, SIRT1 (Sirtuin 1), and others leading to MDM2 auto-ubiquitination and proteasomal degradation (p53 inactivation by deacetylation) [98].

However, with mutated p53, it is difficult to refold and reactivate an inactive tumor suppressor. Two small molecules, MIRA-1 (mutant p53 reactivation and induction of rapid apoptosis) and PRIMA-1 (p53 reactivation and induction of massive apoptosis), can refold mutant p53, restore and stabilize the original DNA-binding domain, and enhance expression of several p53 targets, including Bax (BCL-2-associated X protein), PUMA (p53 upregulated modulator of apoptosis), and Noxa also known as PMAIP1 (phorbol-12-myristate-13-acetate-induced protein 1). In HCC cell lines with mutant p53, PRIMA-1 was demonstrated to exert cytotoxic effects but did not re-establish wild-type DNA binding and transcriptional activities [99].

A second small molecular inhibitor, RETRA (reactivation of transcriptional reporter activity), was shown to increase levels of TAp73 by disrupting an inhibitory TAp73/mutant p53 complex. RETRA disrupts complexes between GOF mutant p53 and TP63/p73, thereby restoring expression of TP63/p73 target genes and tumor inhibition [100].

Further studies are ongoing to clarify the molecular mechanisms that regulate the interaction of the various isoforms of the p53 family and various p53 mutants. Recently, it has been discovered that all members of the p53 family are expressed as a wide range of isoforms. The function of a gene can be switched from tumor suppressor to oncogene depending on the isoform produced. Therefore, the dominant-negative (ΔN) isoforms of p63 and p73, which are important in carcinogenesis and chemoresistance, should be specifically considered. Therefore, selective targeting of different p53 family isoforms may provide the basis for the development of novel therapeutic strategies for HCC and other human cancers [89].

## 3. Emerging Molecular Mechanisms and Therapeutic Vulnerabilities in HCC

### 3.1. Cancer Stem Cell Plasticity and Tumor Progression in HCC

During the past two decades, research on the identification and characterization of liver cancer stem cells (CSCs) has been greatly expanded, and novel strategies for HCC diagnosis and therapy have been developed [101,102]. These studies have highlighted new characteristics of liver CSCs, such as heterogeneity and different immunobiology, that could lead to future investigations and therapeutic strategies [101]. The CSC concept suggests that cancers contain a subpopulation of tumor stem cells that drive tumor growth [103]. This paradigm explains the recurrence of tumors after successful initial chemotherapy and radiation therapy as well as the phenomenon of tumor dormancy and treatment resistance in HCC (as well as other cancers).

CSCs determine the hierarchical organization in organogenesis and tumorigenesis and are responsible for tumor recurrence, metastasis, and chemoresistance. A deeper understanding of the molecular signaling pathways that regulate cellular stemness and hierarchy, as well as the identification of important CSC-specific genes, has led to the rapid development of new diagnostic and therapeutic strategies [104]. Experimental validation of xenotransplantation of recently resected HCC specimens has identified CSC characteristics. CSC markers in HCC include the oval cell marker OV6, CD133, CD90, CD44, CD24, CD13, and EpCAM (epithelial cell adhesion molecule) and Hoechst dye efflux or aldehyde dehydrogenase activities. Some of these CSC markers may support some phenotypes of liver CSCs, such as highly invasive phenotypes and chemoresistance [104].

HCC progression is associated with liver cancer stem cells (LCSCs), which may arise from transformed hepatic progenitor cells or from liver tumor cells that acquire stem-like properties; chronic liver diseases such as NASH (nonalcoholic steatohepatitis) may further promote these processes, although the underlying mechanisms remain incompletely understood. Targeting CSCs to prevent tumorigenesis will likely be beneficial for the design of next generation therapies for HCC. Recently, the effect of CSCs on metastasis, drug tolerance, prognosis, and relapse of HCC has been confirmed [105,106,107]. Two closely related factors, self-renewal and the ability of CSCs to initiate tumors, may be important causes of HCC metastasis. The CSC markers and EMT (epithelial–mesenchymal transition) are closely related to HCC metastasis. This association between unique CSC markers and EMT provides a potential therapeutic angle against HCC metastasis [108]. On the contrary, other studies have shown that CD13 overexpression suppresses metastasis by reducing ROS (reactive oxygen species) via EMT [109]. For instance, Lee et al. (2019) reported that ROS promotes tumor metastasis, which involves migration, invasion, and angiogenesis [110].

Heterogeneity of CSCs is considered to contribute to the molecular and biological heterogeneity of HCC and is associated with poor prognosis. CSC-related markers may also be useful as potential markers for predicting disease outcomes, for example, DKK1 mRNA, which has been investigated as a prognostic marker in HCC. CSCs also contribute to treatment resistance through several mechanisms, including promoting drug efflux, surviving oxidative stress and DNA damage. Several factors, including gene expression, the tumor microenvironment and various signaling pathways, affect the development and maintenance of CSC characteristics. These factors play an important role in the regulation of the CSC phenotype, although the mechanisms are not fully understood [111]. Several oncofetal markers such as CD13, CD133, CD24, CD44, CD90, CACNA2D1 (calcium voltage-gated channel auxiliary subunit alpha2delta 1), DLK1 (delta-like non-canonical Notch ligand 1), EpCam (epithelial cell adhesion molecule), and OV6, have been used to identify CSCs in HCC. Interestingly, some studies showed that some of these liver CSC markers have functional roles in maintaining CSC properties. For example, EpCAM enhances Wnt signaling in both ES and cancer cells, and CD133 expression is necessary for the survival of CD133+ liver CSCs via activation of neurotensin/IL-8/CXCL1 signaling [104]. Furthermore, redox status is modulated by a CD44 variant through the stabilization of xCT (cystine/glutamate antiporter) that protects against oxidative stress in CSCs, and CD13 decreases oxidative stress-induced cell damage after exposure to genotoxic reagents [104]. Thus, most liver CSC markers might be a viable target for liver CSC elimination due to their functional role in maintaining liver CSC characteristics.

CSC markers like CD40 and CD90 are useful to identify and isolate CSC-enriched populations from heterogeneous tumor cells and may also serve as targets for therapeutic intervention [112]. Furthermore, the inhibition of CSCs’ growth and differentiation is also achieved by targeting the blood supply and metabolism of the tumor microenvironment that CSCs rely on. Epigenetic mechanisms, including alterations in histone modifications and DNA methylation, are involved in the regulation of CSC-associated genes and thus are possible therapeutic targets. Several signaling pathways, in particular Wnt/β-catenin, Notch, and STAT3 (signal transducer and activator of transcription 3) maintain CSC properties and therefore serve attractive therapeutic targets. Other approaches include CSC-directed vaccines, activation of resting CSCs to enter the cell cycle, targeted delivery using micro and nanotechnology, promoting CSC differentiation, and improving their response to chemotherapy and radiotherapy [113].

### 3.2. Immune Evasion and the Immunosuppressive Tumor Microenvironment in HCC

The immune-associated cells in the microenvironment greatly affect the development and progression of HCC, and this association is getting more attention. Immune-associated cells can promote or prevent the development of HCC and have a range of other more less well characterized functions [114,115]. Immune escape is a major challenge in HCC treatment. Recent studies have focused on the HCC immune microenvironment to better understand the mechanisms of tumor-induced immunosuppression. These findings have helped identify new therapeutic targets and support the development of different immunotherapy approaches for HCC [116,117,118]. In the tumor microenvironment of HCC, T cells, B cells, macrophages, and hepatic cells interact intricately to initiate cell signaling cascades to evade immune surveillance [114]. High numbers of regulatory T cells (Tregs) are often associated with poorer outcomes, whereas higher levels of CD8+ T cells are linked to a better prognosis. Tregs can suppress the anti-tumor activity of T lymphocytes both in vitro and in vivo [114]. In one study with HCC patients increased numbers of circulating Tregs have been reported, suggesting that these immunosuppressive cells may contribute to disease progression [119]. Immune suppression in HCC is also associated with changes in cytokine production. Levels of immunosuppressive cytokines, including IL (interleukin)-4, IL-5, IL-8, and IL-10, may increase, while immune-activating cytokines such as IL-1, TNF (tumor necrosis factor), and IFN-γ (interferon-γ) may be reduced [120].

In a study of 74 patients with unresectable HCC, higher serum IL-10 levels were associated with poor prognosis and reduced anti-tumor immune activity [121]. Another mechanism of immune escape is the recently discovered programmed cell death-1 (PD-L1/PD-1) immune checkpoint pathway [122]. Immunohistochemical studies of HCC tissues have shown elevated PD-1 and PD-L1 expression, which may promote CD8+ T-cell apoptosis and help tumor cells evade the immune response [123]. Blocking checkpoint molecules that prevent the immune system from attacking tumor cells is now widely considered a successful strategy for the treatment of certain types of solid cancer. Nivolumab, an anti-PD-1 monoclonal antibody, has demonstrated antitumor activity in a report from a phase I/II trial in patients with advanced HCC [124]. A better understanding of liver immunotherapy and immune tolerance may help identify effective immunomodulatory targets for HCC treatment.

### 3.3. Epigenetic Reprogramming and Chromatin Dysregulation in HCC

Epigenetic reprogramming and chromatin dysregulation are common hallmarks of many solid tumors, but HCC shows specific patterns of these changes that are modified by the hepatic environment and the specific etiology of the disease. Epigenetics studies related to heritable traits that are not caused by changes in the DNA sequence that control the structure of chromatin and the accessibility of the transcriptional machinery to the DNA to modulate gene expression and are heavily implicated in HCC and other cancers [125]. HCC is associated with epigenetic changes such as altered expression of micro-RNAs (miRNAs) and lncRNAs, altered histone modification patterns, hypermethylation or hypomethylation of DNA and chromatin remodeling [126]. Specifically, HCC malignancy is strongly linked to abnormalities in DNA methylation and the enzymes that mediate it [127]. There are distinct types of DNA methylation changes in cancer: global hypomethylation of genes and repetitive sequences, which promotes genomic instability and oncogene activation and hypermethylation of the CpGIs in the promoter regions of tumor suppressor genes [128].

Genome-wide DNA methylation profiling has been used in many studies in the last ten years to highlight different methylome patterns in HCC relative to normal tissues. Most of these HCC studies were directed to the identification of significantly hypermethylated genes and amongst them CDKN2A (cyclin-dependent kinase inhibitor 2A), RASSF1 (Ras association domain family member 1), APC and SMAD6 emerged [127,129,130,131,132]; while less studies described hypomethylated genes, such as IGF2, CCL20 and NQO1 [133,134,135]. DNA methylation and histone modification are closely related processes, because inhibitors revert histone modification changes on H3-K4 and H3-K9 codes. Several histone modifications associated with HCC that affect normal cellular activities have been reported [134]. For example, Magerl et al. demonstrated that dimethylation of histone H3 at lysine 4 (H3K4diMe) was poorly expressed in HCC [136], while Shon et al. showed that Patt1 (a GNAT family acetyltransferase) is downregulated in HCC and overexpressed in healthy liver [137]. 1–6% of HCC have somatic mutations that resulted in the silencing of the MLL1-5 (Mixed-Lineage Leukemia 1–5) enzymes that cause H3K4 methylation. Contrary to this, the high H3K4me3 is correlated with the mutations of SMYD3 methyltransferase, leading to a poor prognosis, especially at the early stage of HCC; high H3K4me3 was an independent predictor of poor overall survival (HR = 3.592; 95% CI, 2.302–5.605) [126].

Histone deacetylases (HDACs) are enzymes that are critically involved in the regulation of gene expression by removing the acetyl groups from histones and leading to a more compact structure of DNA and subsequent silencing of genes. There are around 18 HDACs, and the activity is not limited to histones, as it has been documented that HDACs remove acetyl-lysine from a variety of nonhistone proteins such as NF-kB, transcription factors p53, and many others [138]. Individual HDAC overexpression seems to be associated with a poor prognosis in different types of cancer, including HCC. HDAC3 overexpression correlated with early recurrence of HCC after resection and advanced tumor stage. The function of HDACs in HCC should be considered in an isoform- and disease-context-dependent manner rather than generalized across the entire HDAC family. Therefore, the biological and therapeutic effects of HDAC inhibition may depend on the specific HDAC targeted and the stage of liver disease.

**Figure 3:**
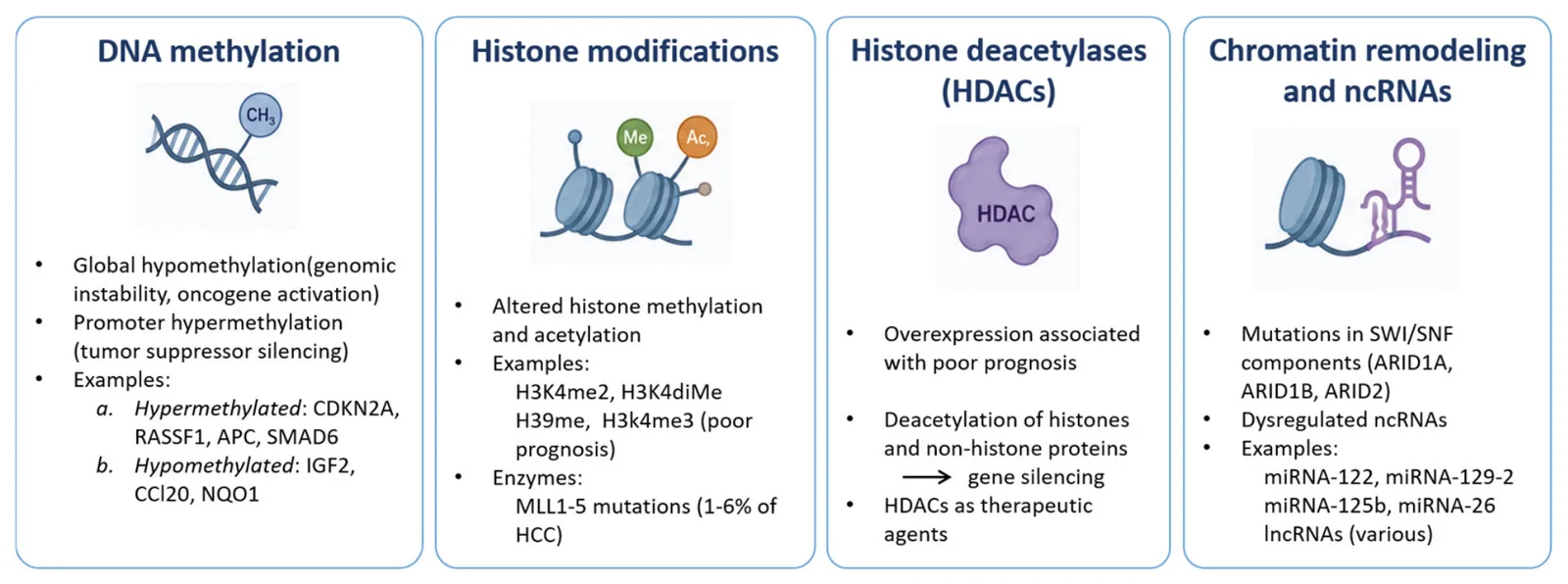
Major epigenetic mechanisms contributing to HCC.

The alterations of multiple pathways by HDAC inhibition could complicate their use for HCC and pleiotropic effects on gene expression should be considered. HDAC inhibition may interfere with gene expression through multiple pathways. Chromatin remodeling plays a role in DNA repair and growth (Figure 3). In one study, the SWI/SNF chromatin remodeling complex was used to demonstrate that the expression of brahma (BRM) was significantly reduced in HCC samples [139]. Components of SWI/SNF-related chromatin remodeling complexes, ARID1A, ARID1B, and ARID2, are mutated in 16.8%, 6.7%, and 5.6% of cases, respectively. Variants of ARID1A, ARID1B, and ARID2 are frequent in alcohol-related and HCV-related HCC, respectively [140,141].

Furthermore, long noncoding RNAs play an important role in different types of tumors, including HCC. LncRNAs function as a scaffold, signal, guide, and decoy at the molecular level. In HCC, miR-129-2 expression is significantly decreased, which has been associated with increased DNA methylation [142]. In addition, miR-122 is downregulated in HCC and regulates several targets, including WNT1, IGF1R, SRF, ADAM10, and cyclin G1 [143]. miRNA-125b and miRNA-26 have been demonstrated to suppress the cell cycle in HCC by specifically targeting oncogenic LIN28B and cyclin D2/cyclin E2, respectively [144,145] (Figure 3).

### 3.4. SRC as Emerging Drivers of HCC

SRC, also known as the nuclear receptor coactivator (NCOA) family, are transcriptional coactivators that regulate gene expression by interacting with nuclear receptors and other transcription factors [146]. The SRC family consists of three members: SRC-1 (NCOA1), SRC-2 (NCOA2), and SRC-3 (NCOA3, also known as AIB1) [147]. Although SRC proteins were initially identified as coactivators of steroid hormone receptors, subsequent studies demonstrated that they participate with other transcription factors in numerous signaling networks involved in cell proliferation, survival, metabolism, migration, and tumor progression [148]. Increasing evidence suggests that dysregulation of SRC family members, particularly SRC-3, contributes to the development and progression of HCC [149,150].

Among the SRC family members, SRC-3 is the most extensively studied in HCC [149,150]. Clinical analyses have shown that SRC-3 is frequently overexpressed in human HCC tissues, with elevated expression detected in approximately 51–68% of tumors [151]. Increased SRC-3 expression has been associated with advanced disease, poor clinical outcomes, and shorter cumulative overall survival in HCC patients. Furthermore, SRC-3 gene amplification has been reported more frequently in metastatic and recurrent HCC than in primary tumors, suggesting a role in tumor progression and aggressiveness [149]. These observations suggest that SRC-3 may represent a potential therapeutic target for modulating sorafenib resistance; however, this possibility remains to be validated clinically in HCC.

**Figure 4:**
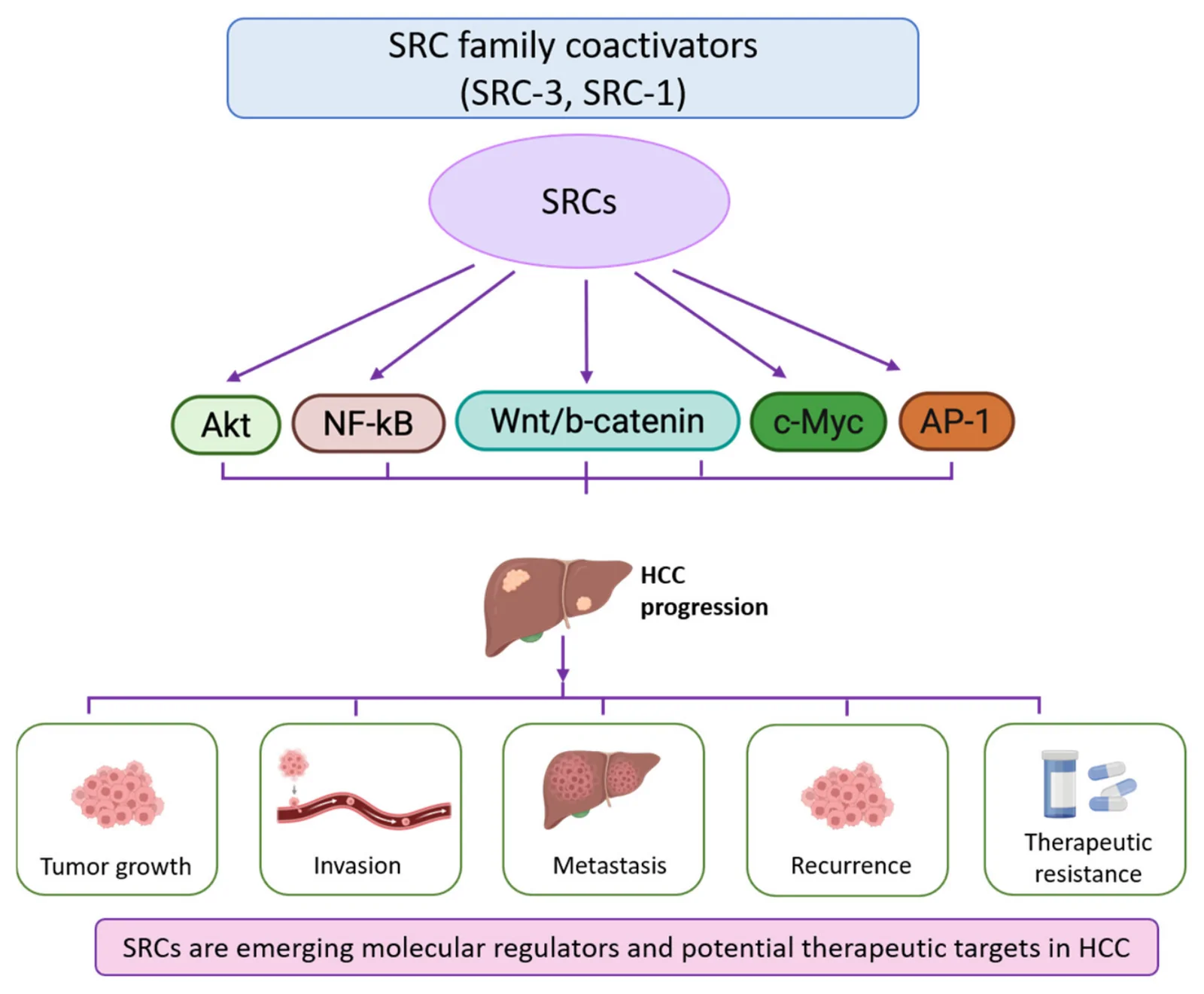
Steroid receptor coactivators (SRCs) as central oncogenic regulators and emerging therapeutic targets in HCC.

Several studies have investigated the biological functions of SRC-3 in HCC cells. Functional analyses demonstrated that SRC-3 promotes HCC cell proliferation, migration, and invasion. Mechanistically, SRC-3 activates multiple oncogenic signaling pathways involved in tumor growth and survival. Xu et al. reported that SRC-3 enhances Akt signaling and promotes cell proliferation in HCC cells [150]. The same study demonstrated that SRC-3 increases expression of MMP-9 (matrix metalloproteinase-9), a protein involved in extracellular matrix remodeling and tumor invasion. Regulation of MMP-9 by SRC-3 was mediated through activation of NF-κB and AP-1 (activator protein 1) transcriptional activity, providing a mechanism by which SRC-3 contributes to the invasive phenotype of liver cancer cells [150]. These findings support a role for SRC-3 as a multifunctional regulator of HCC progression (Figure 4).

HBV infection is a major risk factor for HCC worldwide, and several studies have examined the relationship between viral oncogenic mechanisms and SRC-3. HBV X protein (HBx), a multifunctional viral protein implicated in hepatocarcinogenesis, has been shown to interact with SRC-3. HBx stabilizes SRC-3 protein by disrupting its interaction with the E3 ubiquitin ligase Fbw7α, thereby reducing SRC-3 ubiquitination and degradation. As a result, SRC-3 protein accumulation is increased in HBV-associated HCC cells [152]. In addition, HBx and SRC-3 cooperate to enhance NF-κB-dependent transcriptional activity and promote tumor cell invasiveness [153]. These findings suggest that SRC-3 may serve as an important mediator linking HBV infection to oncogenic signaling pathways involved in HCC progression.

Recent evidence has also linked SRC-3 to therapeutic resistance in HCC [154]. Sorafenib remains an important systemic therapy for advanced HCC; however, acquired resistance frequently limits its clinical benefit. SRC-3 expression has been reported to be increased in sorafenib-resistant HCC models [155]. Mechanistic studies demonstrated that SRC-3 interacts with c-Myc and promotes recruitment of c-Myc to glycolytic gene promoters, thereby enhancing glycolytic metabolism [149]. Through this mechanism, SRC-3 contributes to metabolic reprogramming and promotes resistance to sorafenib treatment. Additional studies have similarly reported that elevated SRC-3 expression is associated with reduced sensitivity to sorafenib. These observations suggest that SRC-3 may represent a potential therapeutic target for overcoming treatment resistance in HCC.

In addition to SRC-3, SRC-1 has also been implicated in HCC progression. Zhao et al. demonstrated that SRC-1 expression is elevated in HCC tissues and is associated with poor prognosis [156]. Functional studies showed that SRC-1 promotes HCC cell growth, migration, and invasion. Importantly, SRC-1 was found to enhance Wnt/β-catenin signaling, a pathway frequently activated in liver cancer [157]. SRC-1 increased β-catenin transcriptional activity and promoted expression of downstream target genes associated with tumor progression [157]. These findings indicate that SRC-1 can function as an important regulator of Wnt/β-catenin-driven hepatocarcinogenesis. Importantly, SRC-1 and SRC-3 were co-overexpressed in 47.5% of HCC specimens, indicating a significant overlap between these two oncogenic coactivators. Simultaneous downregulation of SRC-1 and SRC-3 inhibited HCC cell proliferation more strongly than downregulation of either coactivator alone, suggesting the potential advantages of combined targeting of SRC-1 and SRC-3 in HCCs with SRC-1/SRC-3 co-overexpression.

An important role of SRC in HCC is their ability to connect multiple oncogenic signaling networks rather than acting through a single pathway. SRC-3 has been linked to Akt, NF-κB, AP-1, and c-Myc-dependent transcription, whereas SRC-1 can enhance Wnt/β-catenin signaling [157,158]. Thus, SRCs may help several signaling pathways work together to influence HCC growth, invasion, metabolism, and response to treatment.

Collectively, current evidence identifies SRC family members, particularly SRC-3 and SRC-1, as important contributors to HCC progression. SRC proteins function as transcriptional coactivators that integrate multiple oncogenic pathways, including AKT, NF-κB, AP-1, c-Myc, and Wnt/β-catenin signaling. Their overexpression is associated with tumor growth, invasion, metastasis, recurrence, and therapeutic resistance. Although additional studies are needed to fully define the roles of individual SRC family members in liver cancer, available evidence supports SRCs as emerging molecular regulators of HCC and potential therapeutic targets for future intervention.

Despite these findings, several important questions remain. Most studies in HCC have focused on SRC-3 and, to a lesser extent, SRC-1, whereas the role of SRC-2 remains poorly defined. Much of the mechanistic evidence for SRC-1 and SRC-3 has been obtained from HCC cell lines and experimental models, and their roles across different molecular and etiological subtypes of human HCC remain unclear. It is also not known whether increased SRC expression directly promotes treatment resistance in patients or is primarily associated with a more aggressive tumor phenotype. In addition, although simultaneous downregulation of SRC-1 and SRC-3 produced stronger antiproliferative effects than targeting either coactivator alone in experimental models, the feasibility, specificity, and potential toxicity of targeting SRC requires further investigation. These questions need to be addressed before SRCs can be considered reliable biomarkers or therapeutic targets in HCC.

### 3.5. Progesterone-Mediated Oocyte Maturation Signatures in HCC

The progesterone-mediated oocyte maturation pathway was originally characterized in Xenopus oocytes, where progesterone releases G2/prophase I arrest and promotes entry into meiosis I through activation of the cyclin B–Cdc2/CDK1 complex. This transition is controlled by the phosphatase Cdc25. Cdc25 removes phosphate groups from Cdc2/CDK1 that inhibit its activity and permits the cell to enter M phase [159,160]. Although this pathway was initially defined in the context of oocyte maturation, many of its core components are broadly involved in cell-cycle control and are frequently dysregulated in cancer. Aberrant activation of cell-cycle regulators promotes uncontrolled proliferation, genomic instability, and tumor progression, all of which are hallmarks of hepatocellular carcinoma. Consistent with this concept, pathway-enrichment analyses have identified progesterone-mediated oocyte maturation among the signaling pathways altered in HCC [161].

Jin et al. used a systems biology approach to compare gene expression patterns between HCC patients and healthy controls, identify key genes and altered pathways, and better understand the molecular processes involved in HCC. The authors found that the progesterone-mediated oocyte maturation pathway was closely associated with the onset and progression of HCC and other pathways by using genome-wide relative significance (GWRS) and genome-wide global significance (GWGS), gene ontology (GO) enrichment analysis, pathway analysis, and hub genes identified by the PPI (protein–protein interaction) network [162].

Later, Chen et al. performed a comprehensive analysis of the DEG (differentially expressed gene) between HCC and cirrhotic liver tissue samples by bioinformatics analysis to identify the potential genes and pathways involved in the conversion of HBV-positive liver cirrhosis to HCC. The DEGs were significantly enriched in the KEGG (Kyoto Encyclopedia of Genes and Genomes) pathways of progesterone-mediated oocyte maturation. Further studies are needed to explore the role of the progesterone-mediated oocyte maturation pathway in liver cancer [161].

Importantly, the reported association between the progesterone-mediated oocyte maturation pathway and HCC is based largely on pathway-enrichment analyses and does not demonstrate a progesterone-dependent mechanism in HCC. Many genes included in this pathway, including CDK1, cyclin B, and other cell-cycle regulators, also have important roles in cell division. Therefore, it remains unclear whether enrichment of this pathway represents a specific progesterone-related process or simply reflects changes in cell-cycle regulation in HCC. Experimental studies are needed to determine whether this pathway has a direct role in HCC development or progression.

Representative therapeutic approaches discussed throughout this review are summarized in Table 2 according to their current level of evidence, distinguishing preclinical findings, clinical-trial evidence, and clinically established therapies.

## 4. Conclusions

A comprehensive understanding of the molecular pathways and emerging mechanisms discussed in this review may help identify more effective therapeutic strategies for HCC. Targeting major oncogenic signaling networks together with cancer stem cell-associated pathways, epigenetic regulators, immune-suppressive mechanisms, and steroid receptor coactivators may provide opportunities to improve therapeutic responses and address treatment resistance. Moreover, integrating molecular profiling with pathway-targeted and combination therapies may allow better selection of treatments for molecularly defined groups of HCC patients.

From a translational perspective, molecular changes discussed in this review may also have potential value as biomarkers for prognosis, patient stratification, and treatment response. For example, changes in major signaling pathways, cancer stem cell-associated markers, and SRC expression have been associated with aggressive disease, poor prognosis, or therapeutic resistance. However, many of these candidate biomarkers have not yet been validated for routine clinical use. An important next step will be to distinguish prognostic biomarkers that are associated with disease outcome from predictive biomarkers that can identify patients more likely to respond to a specific therapy.

However, several limitations in the current literature should be considered. HCC is highly heterogeneous, and the importance of individual signaling pathways may vary according to tumor genotype, etiology, disease stage, and the tumor microenvironment. In addition, strong mechanistic findings from experimental models have not always translated into clinical benefit, as illustrated by the limited efficacy of several pathway-specific inhibitors. The evidence supporting some emerging mechanisms is even less complete. The roles of individual SRC family members across different HCC subtypes remain incompletely defined, and the proposed involvement of progesterone-mediated oocyte maturation signaling is currently supported mainly by bioinformatic studies. Future studies should therefore focus on functional validation of these emerging mechanisms, identification of biomarkers that define pathway-dependent tumors, and determination of which pathway-targeted combinations are effective in specific molecular subtypes of HCC.

## Figures and Tables

**Table 1:** Therapeutic inhibitors targeting the MAPK/ERK signaling pathway in hepatocellular carcinoma (HCC).

Inhibitor	Primary Target(s)	Mechanism/Role in HCC
Sorafenib	RAF, VEGFR2, VEGFR3, PDGFR-β, KIT	Multikinase inhibitor that suppresses RAF/MEK/ERK signaling and tumor angiogenesis; first systemic therapy approved for advanced HCC
Lenvatinib	VEGFR1-3, FGFR1-4, PDGFRα, RET, KIT	Inhibits receptor tyrosine kinases upstream of MAPK/ERK signaling and reduces angiogenesis
Regorafenib	RAF-1, BRAF, VEGFR1-3, TIE2, PDGFR, FGFR	Multikinase inhibitor approved as second-line therapy after sorafenib; suppresses MAPK signaling and tumor angiogenesis
Cabozantinib	MET, VEGFR2, RET, AXL	Inhibits multiple oncogenic kinases that activate downstream MAPK/ERK signaling
Sunitinib	VEGFR, PDGFR, KIT, FLT3	Multikinase inhibitor evaluated in HCC but showed no survival advantage over sorafenib
Erlotinib	EGFR	Blocks EGFR-mediated activation of the RAS/RAF/MEK/ERK pathway; demonstrated limited clinical activity in HCC
Selumetinib	MEK1/2	Selective MEK inhibitor that directly targets the MAPK/ERK cascade; evaluated in advanced HCC
Refametinib	MEK1/2	Selective MEK inhibitor investigated in HCC, particularly in combination strategies
Trametinib	MEK1/2	Potent MEK inhibitor that blocks ERK activation; primarily evaluated in preclinical and early clinical studies relevant to HCC

**Table 2:** Therapeutic agents targeting major signaling pathways in hepatocellular carcinoma.

Pathway/Target	Agent	Evidence Level in HCC	Main Finding/Current Status
MAPK/ERK	Sorafenib	Clinically established	Multikinase inhibitor targeting RAF and angiogenic kinases [163]
	Regorafenib	Clinically established	Multikinase inhibitors including RAF-1/BRAF [163]
	Selumetinib	Phase II clinical trial	Selective MEK1/2 inhibitor evaluated in advanced HCC [164]
	Refametinib	Phase II clinical trials	MEK inhibitor evaluated alone and with sorafenib in RAS-mutant HCC [163]
Wnt/β-catenin	E7386	Phase I clinical trial	CBP/β-catenin interaction inhibitor [165]
	PRI-724	Preclinical HCC evidence	Inhibits CBP/β-catenin transcription [166]
	ICG-001	Preclinical HCC evidence	Blocks β-catenin–CBP interaction [167]
PI3K/AKT/mTOR	Everolimus	Phase III clinical trial—negative	mTOR inhibitor; failed to improve overall survival after sorafenib failure [58]
	Temsirolimus	Phase I/II clinical trial	mTOR inhibitor evaluated in unresectable HCC [168]
	Sirolimus	Phase II clinical evidence	mTOR inhibitor evaluated in treatment-naïve advanced HCC [169]
Hippo-YAP/TAZ	Verteporfin	Preclinical HCC evidence	YAP-targeting approach that suppresses HCC growth in experimental models [170]
	VT104	Preclinical HCC evidence	Pan-TEAD inhibitor that reduced tumor growth in experimental HCC models [171]
Cell cycle/CDKs	Palbociclib	Preclinical HCC evidence	CDK4/6 inhibitor that restricted growth of RB1-proficient HCC models [172]
	Dinaciclib	Preclinical HCC evidence	Inhibits CDK1/2/5/9 [173]
	AZD4573	Preclinical HCC evidence	Selective CDK9 inhibitor [174]
p53 pathway	Recombinant adenovirus-p53 (rAd-p53)	Clinical evidence, not established standard therapy	rAd-p53 combined with TACE showed improved OS and PFS versus TACE alone [95]
	Nutlin-3	Preclinical HCC evidence	MDM2 antagonist that activates p53/p73 signaling [175]
	PRIMA-1	Preclinical HCC evidence	Mutant-p53-targeting compound with cytotoxic and p53-modulating activity [176]
SRC	Bufalin	Preclinical HCC evidence	Reduce SRC-1 expression and inhibit HCC metastatic properties [177]
	SRC-1/SRC-3 targeting	Experimental/genetic evidence	Knockdown of SRC-1 or SRC-3 suppresses HCC growth [157]

## Data Availability

No new data were generated or analyzed in this study. Data sharing is not applicable to this article.
